# De novo super-early progeny in interspecific crosses *Pisum sativum* L. × *P. fulvum* Sibth. et Sm

**DOI:** 10.1038/s41598-021-99284-y

**Published:** 2021-10-05

**Authors:** Hatice Sari, Duygu Sari, Tuba Eker, Cengiz Toker

**Affiliations:** Department of Field Crops, Faculty of Agriculture, Akdeniz University, Antalya, 07070 Turkey

**Keywords:** Plant breeding, Plant hybridization, Abiotic, Attribution, Genetics, Plant sciences

## Abstract

Earliness in crop plants has a crucial role in avoiding the stress of drought and heat, which are the most important challenging stressors in crop production and are predicted to increase in the near future due to global warming. Furthermore, it provides a guarantee of vegetable production in the short growing season of agricultural lands in the northern hemisphere and at high altitudes. The growing human population needs super early plant cultivars for these agricultural lands to meet future global demands. This study examined de novo super-early progeny, referred to as much earlier than that of the earlier parent, which flowered in 13–17 days and pod setting in 18–29 days after germination, discovered in F_2_ and studied up to F_5_ derived from interspecific crosses between garden pea (*P. sativum* L.) and the most distant relative of pea (*P. fulvum* Sibth. et Sm.). De novo super-early progeny were found to be earlier by about one month than *P. sativum* and two months than *P. fulvum* under short day conditions in the F_5_ population. In respect of days to flowering and pod setting, de novo super-early progeny had a relatively high level of narrow sense heritability (*h*^2^ = 82% and 80%, respectively), indicating that the selections for earliness in segregating populations was effective for improvement of extreme early maturing varieties. De novo super-early progeny could be grown under heat stress conditions due to the escape ability. Vegetable types were not only high yielding but also free of any known undesirable traits from the wild species, such as pod dehiscence and non-uniform maturity. It could be considered complementary to “speed breeding”, possibly obtaining more than six generations per year in a suitable climate chamber. Not only de novo super-early progeny but also transgressive segregation for agro-morphological traits can be created via interspecific crosses between *P. sativum* and *P. fulvum*, a precious unopened treasure in the second gene pool. Useful progeny obtained from crossing wild species with cultivated species reveal the importance of wild species.

## Introduction

*Pisum sativum* L. is the second most important plant at the point of production among grain legumes with 21.8 million tons of vegetable pea and 14.2 million tons of dry pea in 2019 in the world^1^. Wide areas of usage of pea and high nutritional value make it important. Green pea is harvested before the seed is mature for the fresh or canned market^2^ and fresh pods are consumed as vegetables^3,4^. Pea is not only used as dry, frozen and in animal feed^5^, but is also of great importance as a rotation crop^6,7^ since it improves soil microbial diversity, protects soil water, and enriches the soil organic matter content^8^. Pea, like other legumes, fixes atmospheric nitrogen from the air into the soil. Nodules in pea roots transform atmospheric nitrogen (N_2_) to ammonia (NH_3_) with the bacteria *Rhizobium leguminosarum*^6,9^. Pea is a high-quality source of vegetable nutrition because of its high levels of digestible protein, balanced amino acids^2^, B vitamins and dietary fiber. According to the above-mentioned special characteristics, pea is one of the major crops required to meet the food needs of the growing human population with its high-quality nutritional content.

It is thought that the long generation time of most plant species will create a bottleneck in meeting future food demands and in the application of breeding research^10^. Such needs have increased the need for new technologies such as speed breeding (SB). In the speed breeding procedure, it is aimed for plants to produce more than one generation in one year by growing them under controlled conditions for 22 h light and 2 h dark^10,11^. It has been reported that 6 generations of wheat (*Triticum aestivum* and *Triticum durum*), barley (*Hordeum vulgare*), chickpea (*Cicer arietinum*) and pea (*P. sativum*) and 4 generations of canola (*Brassica napus*) have been obtained in one year using speed breeding^10,11^. Speed breeding allows the use of promising technology, but due to the high costs, this technology is not available everywhere. Therefore, it is necessary to develop genetically early varieties, which are cheaper and more easily accessible.

Earliness, early maturity and yield are the important traits in pea, which determine the pea variety selected by farmers. Flowering time plays a major role in the adaptation of pea to different environments, especially in regions where growth is limited. Wide variations have been reported for the flowering time of pea^12–14^. It has been reported to vary from 140 to 220 days during the growing season in pea-growing regions of Sweden^15^. Earliness is not only crucial to avoid end of season frost^16^ but is also a substantial trait to increase pea productivity by avoiding drought and heat^17,18^. More than 20 loci have been determined in pea related to flowering time and inflorescence improvement. Flowering time is controlled by five major loci in the *Pisum* L. The late flowering (*Lf*) locus^19^ prevents flowering in long and short days^20,21^, but there are numerous allelic variants of *Lf* with both naturally arising and induced mutant alleles. It has been reported that in genotypes with the *Lf* gene, extreme earliness occurs when it is deleted or inactivated by nonsense mutations^21,22^. Recessive alleles at the high response (*Hr*) locus encourage early flowering in short days and reduce the photoperiod response, while the sterile nodes (*Sn*) locus confers a response to the photoperiod^19,23,24^. Dominant alleles at the early (*E*) locus^24^ induce early flowering in some genetic backgrounds^14,25–27^, but this effect may show a complicated interaction with other loci^28,29^. Allelic differences in *Lf*, *Hr*, *Sn* and *E* loci interact, resulting in a very wide range of flowering times of plants in non-inductive conditions^14,19,22,24,28^. The Die Neutralis (*Dne*) locus reduces the photoperiod response like the *Hr* locus, and encourages early flowering in short days^24,27,29,30^. Previous studies have examined the genetics of flowering, QTL (Quantitative trait loci) studies related to flowering, and inheritance of days to flowering (earliness) in the genus *Pisum* L.^12–14,19,21,22,24,27,29–33^. However, the variations including earliness, inheritance of earliness and transgressive segregations, coined as the presence of progeny with values greater or less than the values of their parents in segregated generations^34–36^, and de novo super-early progeny (referred to as progeny with flowering or pod setting and maturity much earlier than the early parent in segregating generations) derived from interspecific crosses between *P. sativum* and *P. fulvum* have not been studied according to the available literature. Therefore, the purpose of this study was (1) to select de novo super-early progeny, (2) to reveal the narrow sense heritability of days to flowering, days to pod setting, and important agro-morphological traits in F_2_ and F_3_ populations, and (3) to determine the transgressive segregations to select superior lines for important agro-morphological traits in F_2_ and F_5_ populations.

## Results

**Figure 1:**
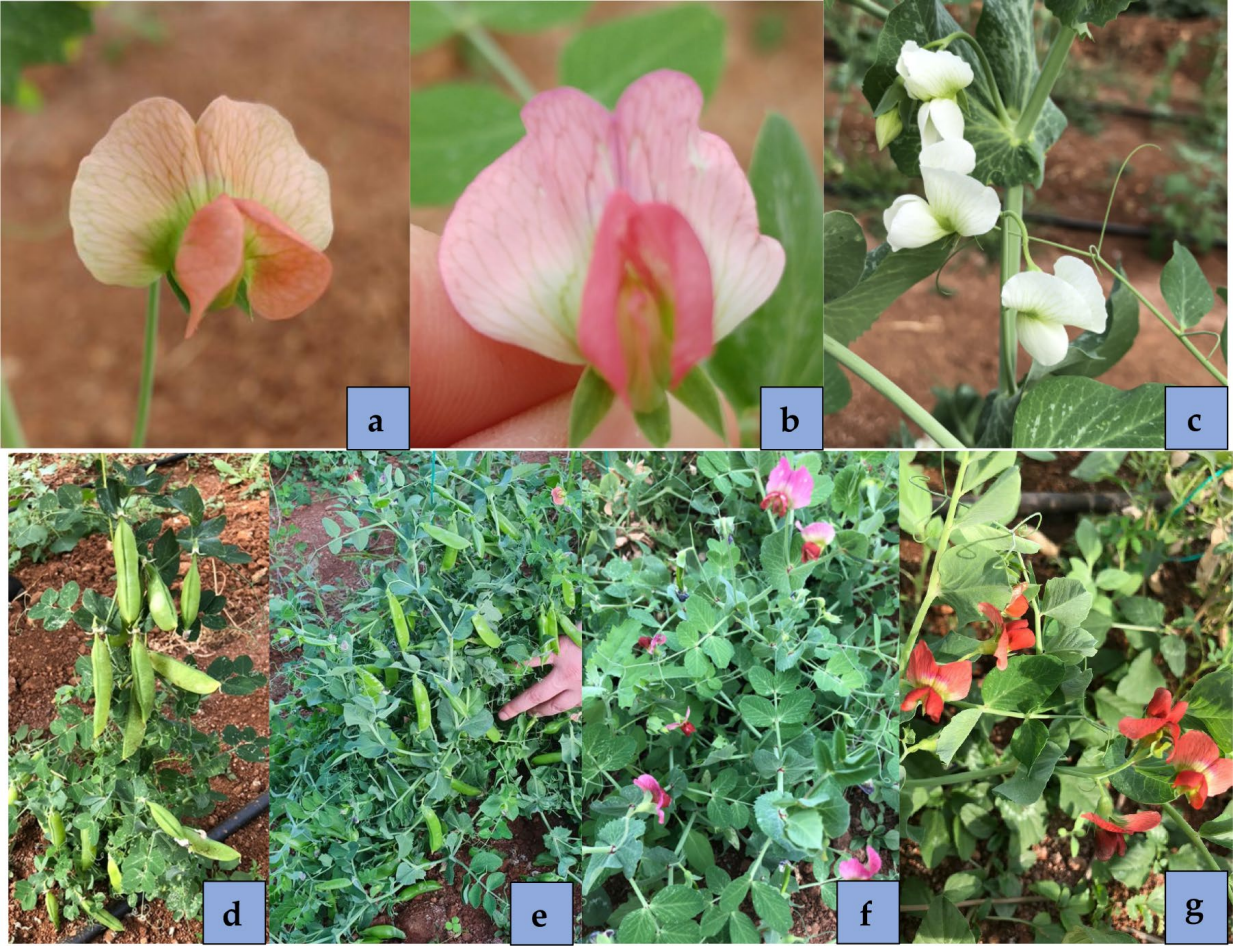
Orange (**a**), fuchsia (**b**) and white (**c**) flower colors in F_2_ population derived from interspecific crosses *P. sativum* (ACP 20) × *P. fulvum* (AWP 600). Super early progeny for fresh seeds (**d**), Super early progeny for vegetables (**e**), View of the lines (**f**, **g**).

The seed coat color (testa) and surface of *P. sativum* (ACP 20) were seen as yellowish cream and wrinkled, while these traits were recorded as black and smooth in *P. fulvum* (AWP 600) (Table 1). After pollination, the seed coat color and surface were found to be black and smooth in reciprocal interspecific crosses between *P. sativum* × *P. fulvum* and *P. fulvum* × *P. sativum*. Both F_1_ plants derived from reciprocal interspecific crosses had black seed coat and smooth seed coat. The flower color of F_1_ plants was orange, while *P. sativum* and *P. fulvum* had white and orange flower colors, respectively. The flower color in the plants derived from the F_2_ population were separated as three distinct categories of orange, fuchsia and white colors (Fig. 1). Segregations for flower color were fit well to 12 (orange) :3 (fuchsia) :1 (white), dominant epistasis (Table 2).

**Table 1:** Some agro-morphological and salient traits of parents used in interspecific crosses *P. sativum* × *P. fulvum.*

Species	Parents	Flower color	Seed color	Seed shape	100-seed weight (g)	Resistance to a/biotic stresses
*P. sativum*	ACP 20			Wrinkled	42.8	Powdery mildew
*P. fulvum*	AWP 600			Smooth	5.4	Powdery mildew, seed beetle

**Table 2:** Chi-square analysis for flower color in interspecific crosses *P. sativum* × *P. fulvum.*

Crosses	Flower color (F_1_)	Flower colors (F_2_)	Estimated ratio	No of plants		${\chi }^{2}$	*P*
				Observed	Estimated		
ACP 20 × AWP 600	Orange	Orange	12	77	86.2	7.73	0.25–0.1
		Fuchsia	3	24	21.5		
		White	1	14	7.2		

### Heterosis in F_1_ progeny

**Figure 2:**
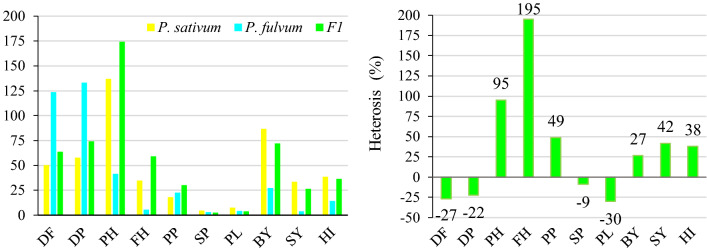
Comparison of agro-morphological traits in F_1_ derived from interspecific crosses *P. sativum* × *P. fulvum* and their parents (left) and heterosis values (%) of F_1_ plants (right). (DF is days to flowering, DP is days to pod setting, PH is plant height, FH is first pod height, PP is number of pods per plant, SP is number of seeds per plant, PL is pod length, BY is biological yield, SY is seed yield, HI is harvest index).

For days to flowering and days to pod setting traits, the F_1_ plants derived from interspecific crosses *P. sativum* × *P. fulvum* had negative average heterosis values of − 27% and − 22%, respectively (Fig. 2). Plant height had a heterosis value of 95%, while the first pod height had a heterosis of 195%. Heterosis for the pods per plant, the seeds per pod, pod length and biological yield traits was found to be 49%, − 9%, − 30%, and 27%, respectively. For the seed yield per plant, considerable heterosis was detected at 42%, while heterosis for the harvest index was 38% (Fig. 2).

### Transgressive segregation for agro-morphological traits in F_2_ and F_3_ populations

In the F_2_ population, 121 individual plants from four F_1_ plants derived from interspecific crosses *P. sativum* × *P. fulvum* were grown in the same glasshouse. Plant height for *P. sativum* and *P. fulvum* was 137 cm and 41.3 cm, respectively (Table 3). Plant height for F_2_ and F_3_ populations was determined to be 16–244 cm and 7–252 cm, respectively. The number of pods per plant was 18 for *P. sativum* and 22.7 for *P. fulvum*, between 7 and 81 in F_2,_ and 1 and 68 in F_3_ population. The number of seeds per pod in *P. sativum* and *fulvum* was 4.7 and 3, respectively, and 1–4.7 in F_2_ and 1–5 in F_3_ population. The biological yield was 86.5 g for *P. sativum* and 27. 2 for *P. fulvum*, the seed yield was 33.4 g for *P. sativum* and 3.9 for *P. fulvum*, and the harvest index was 38.3% for *P. sativum* and 14.4% for *P. fulvum.* Biological yield was 26–237 g for F_2_ population and 4–243 g for F_3_ population, seed yield was 10–83 g for F_2_ population and 4.2–79.8 g for F_3_ population (Table 3).

**Table 3:** Minimum (Min) and maximum (Max) values, means ($\overline{\mathrm{X} }$) $\pm$ standard errors (${\mathrm{S}}_{\overline{\mathrm{X}} }$) and narrow-sense heritability (${h}^{2}$) for agro-morphological traits in parents and progeny derived from interspecific crosses *P. sativum* × *P. fulvum.*

Traits	ACP 20		AWP 600		F_1_		F_2_		F_3_		*h*^2^ (%)
	Min–Max	$\overline{\mathrm{X }}\pm {\mathrm{S} }_{\overline{\mathrm{X}} }$	Min–Max	$\overline{\mathrm{X }}\pm {\mathrm{S} }_{\overline{\mathrm{X}} }$	Min–Max	$\overline{\mathrm{X }}\pm {\mathrm{S} }_{\overline{\mathrm{X}} }$	Min–Max	$\overline{\mathrm{X }}\pm {\mathrm{S} }_{\overline{\mathrm{X}} }$	Min–Max	$\overline{\mathrm{X }}\pm {\mathrm{S} }_{\overline{\mathrm{X}} }$	
Days to flowering (DF)	48.0–52.0	50.3 ± 1.2	119.0–127.0	123.7 ± 2.4	63.0–64.0	63.5 ± 0.3	17.0–96.0	58.6 ± 1.9	13.0–180.0	97.4 ± 4.7	82
Days to podding (DP)	55.0–60.0	57.7 ± 1.4	128.0–139.0	133.3 ± 3.2	73.0–75.0	74.3 ± 0.5	29.0–103.0	68.6 ± 1.9	18.0–188.0	103.2 ± 4.7	80
Plant height (PH)	134.0–142.0	137.0 ± 2.5	39.0–43.0	41.3 ± 1.2	167.0–178.0	174.3 ± 2.5	16.0–244.0	129.0 ± 6.4	7.0–252.0	94.5 ± 6.3	45
First pod height (FH)	33.0–36.0	34.7 ± 0.9	5.0–6.0	5.3 ± 0.3	51.0–64.0	59.0 ± 2.9	2.0–102.0	36.9 ± 2.7	1.0–92.0	22.7 ± 1.9	45
No of pods per plant (PP)	16.0–20.0	18.0 ± 1.1	21.0–24.0	22.7 ± 0.9	29.0–32.0	30.3 ± 0.6	7.0–81.0	41.3 ± 2.0	1.0–68.0	18.5 ± 1.1	33
No of seed per pod (SP)	4.0–5.0	4.7 ± 0.3	3.0–3.0	3.0 ± 0.0	2.0–3.0	2.5 ± 0.3	1.0–4.7	1.9 ± 0.1	1.0–5.0	2.4 ± 0.1	50
Pod length (PL)	7.0–8.0	7.3 ± 0.3	4.0–4.5	4.2 ± 0.2	3.7–4.3	3.9 ± 0.2	2.7–7.7	4.7 ± 0.1	1.0–8.0	4.3 ± 0.1	36
Biological yield (BY)	79.8–91.3	86.5 ± 3.4	26.7–27.9	27.2 ± 0.3	67.0–78.0	72.0 ± 2.7	26.0–237.0	124.5 ± 6.1	4.0–243.0	75.0 ± 4.9	37
Seed yield (SY)	24.8–40.2	33.4 ± 4.5	3.7–4.3	3.9 ± 0.2	23.0–30.0	26.3 ± 1.5	10.0–83.0	35.8 ± 1.8	4.2–79.8	14.4 ± 1.8	28
Harvest index (HI)	31.0–44.0	38.3 ± 3.8	13.9–15.4	14.4 ± 0.5	33.8–38.5	36.4 ± 1.0	8.7–69.7	31.8 ± 1.5	2.8–65.7	22.6 ± 0.9	16

### De novo super-early progeny

**Figure 3:**
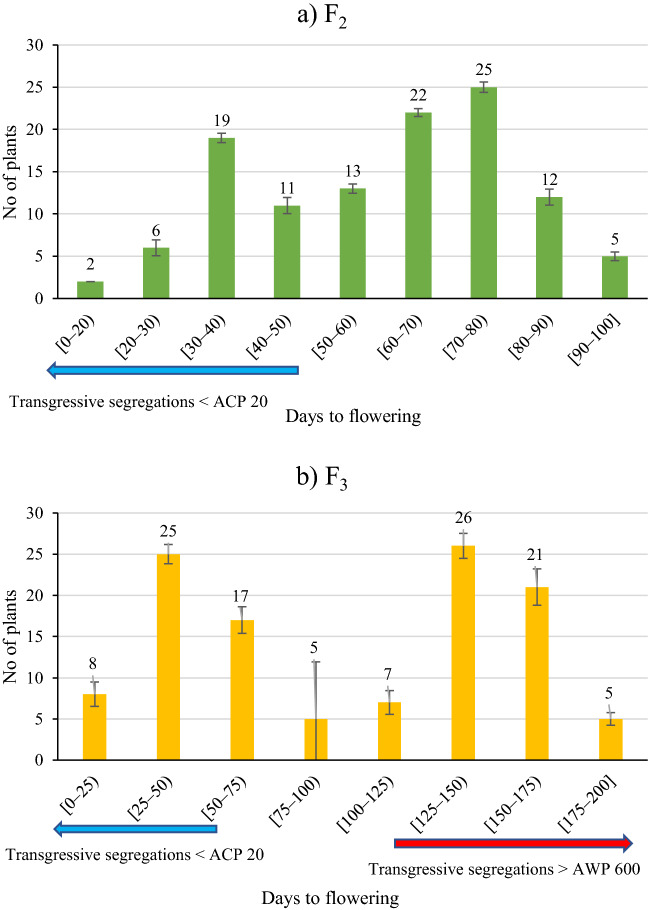
Frequency distribution of days to flowering in F_2_ (**a**) and F_3_ (**b**) populations. Transgressive segregations were shown by arrows (blue and red colors). De novo super-early progeny are earlier than the earliest parent (Blue color).

The number of days to flowering was recorded as 50.3 days for *P. sativum* and 123.7 days for *P. fulvum*, while the de novo super-early progeny flowered 17 days after germination in F_2_ population and after 13 days in F_3_ population. The number of days to pod setting was 57.7 days for *P. sativum* and 133.3 days for *P. fulvum*. The de novo super-early progeny formed pods in 29 days in F_2_ population and in 18 days in F_3_ population (Fig. 3 and Table 3).

Days to flowering in F_2_ and F_3_ populations was found to be appropriate bimodal distribution (Fig. 3a,b) as early (earlier than the domesticated pea) and late (later than the domesticated pea) plants. Distribution on days to flowering was found to be fit well to 9 (late progeny):7 (early progeny) segregation ratio in F_2_ population. Days to flowering seems to be controlled primarily by two genes having duplicate recessive epistasis under the short day conditions.

**Figure 4:**
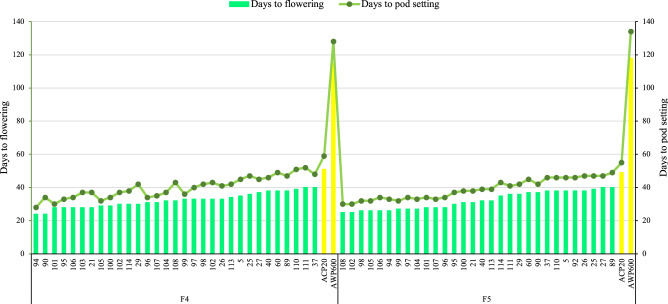
Days to flowering and days to pod setting of parents and de novo super-early progeny in F_4_ and F_5_ populations derived from interspecific crosses *P. sativum* × *P. fulvum*.

While days to flowering of the de novo super early progeny in F_4_ were between 24 and 34 days, days to pod setting varied between 28 and 48 days. Days to flowering of de novo super early progeny in F_5_ was determined to be between 25 and 40 days and days to pod setting was between 30 and 47 days (Fig. 4). The de novo super early progeny occurred especially in the F_2_ and F_3_ populations, but not in the parents. De novo super early progeny flowered after 18 days in F_2_ population (Fig. 3a), and when segregated for earliness in the later generations, flowered after 25 days in F_5_ (Fig. 4). Some of the de novo super early progeny were selected for fresh green pod and seed in the F_5_ population (Fig. 1d,e).

### Heritability

Narrow-sense heritability (${h}^{2}$) values were highest for days to flowering (82%) and days to pod setting (80%) (Table 3). While the narrow-sense heritability of the number of seeds per pod was 50, the plant height and the first pod height were 45. The heritability of biological yield, pod length, number of pods per plant, and seed yield were found to be 37%, 36%, 33% and 28%, respectively. Harvest index had the lowest narrow-sense heritability at 16% (Table 3).

### Relationships between correlated traits and lines

**Figure 5:**
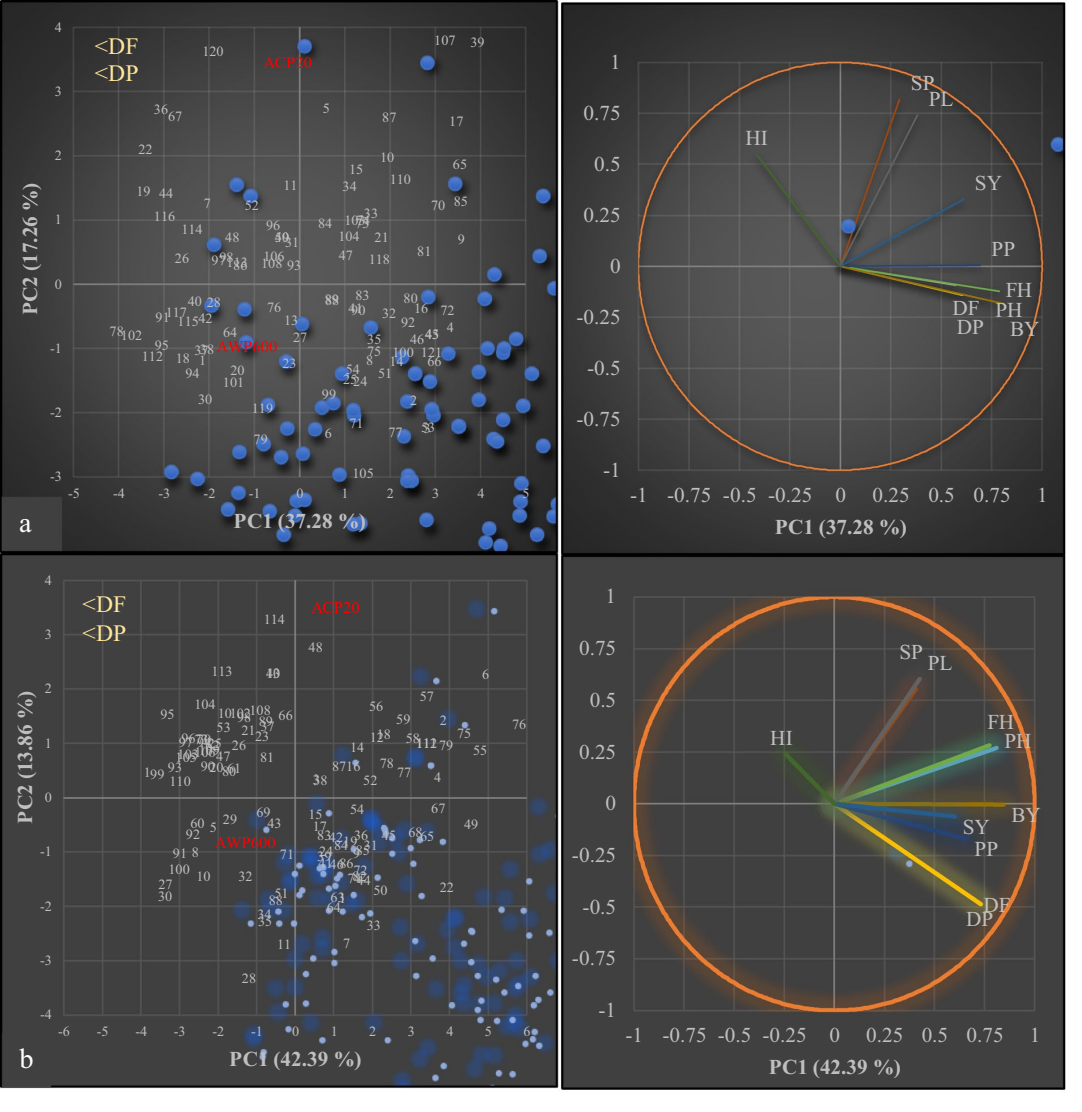
Principal component analyses (PCAs) for in F_2_ (**a**) and F_3_ (**b**) populations derived from interspecific crosses *P. sativum* × *P. fulvum*. Each blue dot represents a progeny in F_2_ and F_3_ populations. (DF is days to flowering, DP is days to pod setting, PH is plant height, FH is first pod height, PP is number of pods per plant, SP is number of seeds per plant, PL is pod length, BY is biological yield, SY is seed yield, HI is harvest index).

Eigenvalues of the principal component analyses (PCAs) in the F_2_ population, were found to be greater than 1 for four components. However, the diagram explained 54.5% of the total variance with two components (Fig. 5a). PC1 was closely related to plant height (PH), first pod height (FH), number of pods per plant (PP) and biological yield (BY) at 37.3%. PC2 was related to the number of seeds per pod (SP) and pod length (PL), representing 17.2% of the total variance. The third component was related to days to flowering (DF) and days to pod setting (DP) and explained 16.2% of the total variance. In addition, the progeny in the upper left part of the PCA diagram were those with earlier flowering and pod setting than the others (Fig. 5a). Considering the PCA results of the F_3_ population, it was divided into two components. The first component (PC1) was related to almost all traits (DF, DP, PH, FH, PP, BY and SY) with a variance of 42.39%. PC2 represented 13.86% of the total variance with SP and PL traits (Fig. 5b). As in the F_2_ population, de novo super-early progeny in the F_3_ population were found in the upper left part of the PCA diagram (Fig. 5a,b).

### Direct and indirect effects on agro-morphological traits over days to flowering

To examine the direct and indirect effects of agro-morphological traits on days to flowering, path (p) analysis was performed on the F_3_ population derived from inter-specific crosses *P. sativum* × *P. fulvum* (Table 4). Days to pod setting and first pod height had statistically significant (*P* < 0.05) direct effects on days to flowering, and the coefficients were *p* = 0.999* and *p* = 0.018*, respectively. In addition, plant height with *p* = − 0.021* had negative direct effect on days to flowering. Plant height (*p* = − 0.498*), first plant height (*p* = 0.655*), pods per plant (*p* = 0.303*) and biological yield (*p* = -0.288*) had significant indirect effect on days to flowering over days to pod setting (Table 4).

**Table 4:** Path coefficient showing direct (bold) and indirect effects for agro-morphological traits on days to flowering (DF) in F_3_ population advanced from interspecific crosses *P. sativum* × *P. fulvum.*

Traits	DP	PH	FH	PP	SP	PL	BY	SY	HI
DP	0.999*	− 0.117	0.165	0.190	− 0.124	0.132	0.139	− 0.058	− 0.074
PH	− 0.498*	− 0.021*	0.840*	0.674*	− 0.178	0.447	0.295	− 0.390	− 0.307
FH	0.655*	0.786*	0.018*	− 0.642*	0.290	− 0.101	0.208	0.103	0.253
PP	0.303*	0.254*	− 0.258*	**0.003**	0.321*	− 0.313*	0.164	0.409*	− 0.260
SP	− 0.103	− 0.035	0.061	0.167	− **0.001**	0.326*	− 0.026	0.036	0.004
PL	0.118	0.094	− 0.023	− 0.175	0.350*	**0.003**	− 0.035	0.143	0.108
BY	0.288*	0.144	0.109	0.213	− 0.065	− 0.082	**0.001**	0.551*	0.087
SY	− 0.075	− 0.120	0.034	0.334	0.057	0.209	0.347*	− **0.002**	− 0.052
HI	− 0.055	− 0.053	0.047	− 0.120	0.004	0.089	0.031	− 0.029	**0.004**

DP is days to pod setting, PH is plant height, FH is first pod height, PP is number of pods per plant, SP is number of seeds per plant, PL is pod length, BY is biological yield, SY is seed yield, HI is harvest index. * Indicates significant relationships at *P* < 0.05.

## Discussion

Wild pea species are not only useful genetic resources for resistance to different a/biotic stresses^37–52^, but they also possess nutritional value for food and feed, desirable agronomic traits and advantages in nitrogen fixation^53^. Progeny have successfully been produced by interspecific crosses when *P. fulvum* was used as a pollen donor^37,41,45,54^. *P. fulvum* was reported in the second gene pool of the genus *Pisum*^55^. According to the available literature, reciprocal interspecific crosses were reported by Kosterin et al.^56^ as in the present study.

After hybridization, seed coat color and surface of the seed coat were recorded as black and round inside pods in *P. sativum,* mother or pollen receiver plant when *P. fulvum* species was used as a pollinator. A similar color and surface were found in F_1_ plants indicating that black seed color and round seed surface were dominant over their counterparts. Gregor Johann Mendel, known as the father of genetics, reported a similar finding on seed coat surface in intraspecific crosses in pea crop 156 years ago^57^. The flower color of both F_1_ plants was orange (Fig. 1) indicating that orange flower color was dominant over white flower color as reported by Kosterin et al.^56^. Kosterin et al.^56^ reported that flower color was quantitatively inherited in an F_2_ segregation population derived from reciprocal interspecific crosses *P. sativum* × *P. fulvum* and *P. fulvum* × *P. sativum.* However, flower color in one way interspecific crosses *P. sativum* × *P. fulvum* was segregated in the F_2_ population as three distinct categories with 12 (orange): 3 (fuchsia): 1(white colors) (Fig. 1a–c) segregation ratio (Table 2) inferring as dominant epistasis. Prior to the present study, interspecific crosses *P. sativum* × *P. fulvum* were reported in several studies^37,41,45,48,56,58–60^, while hybrids *P. fulvum* × *P. sativum* have been introduced in only research study to date^56^.

Negative average heterosis was obtained for days to flowering (− 27%) and days to pod setting (− 22.2%) traits (Fig. 2). Guindon et al.^61^ similarly found negative heterosis for days to flowering, as 0.48% heterosis for plant height, and 27% for the number of pods. In interspecific crosses, average heterosis was recorded for plant height (95%), pods per plant (49%), seed yield (42%) and harvest index (38%) that were important for yield (Fig. 2). However, traits including seeds per pod (− 9%) and pod length (− 29.8%) had negative average heterosis (Fig. 2). Sarawat et al.^62^ reported that heterosis was negative for seeds per pod (− 1.4%) and 100-seed weight (− 2.1%), while days to flowering (earliness) at 1.4%, plant height at 19.2%, seed yield at 30.8%, pods per plant at 38.5% and harvest index at 0.6% had positive heterosis.

Not only in the F_2_ population but also in the F_3_ population, transgressive segregations were investigated for agro-morphological traits. However, differences between maximum and minimum values for some agro-morphological traits were declined in the F_3_ population indicating that transgressive segregations were reduced from F_2_ to F_3_ population (Table 3). The term of transgressive segregation was coined as a phenomenon specific to segregating generations and refers to the fraction of progeny that exceeds the parents in either a negative or positive direction^34–36^. This phenomenon is similar to heterosis in first-generation hybrids. Rieseberg et al.^36^ defined the creation of transgressive segregations as: (1) mutation frequency in segregating populations; (2) reduced developmental stability; (3) non-additive allelic effects between loci or epistasis; (4) non-additive allelic effects within a locus or overdominance; (5) the unmasking of some recessive alleles that are generally heterozygous in the parents; (6) variation in chromosome number; and (7) the complementary action of additive alleles that are dispersed between the parents. In the present study, except for mutation frequency and variation in chromosome number, five reasons for transgressive segregations in F_2_ and F_3_ populations could be considered. Flowering time in pea has been considered to be two genetic control systems as a result of an increase in recessive genes for early flowering, and dominant genes for late flowering^63^. Earliness in pea crosses was related with additive and non-additive genetic effects^64,65^. Transgressive segregations in segregating generations in interspecific crosses in *Cicer* species have been previously outlined^66,67^. One of the main objectives of most breeding studies is to increase yield, but it is a challenge for breeders to achieve an increase because the yield is affected by genes and environments due to its polygenic nature. Therefore, it is important to benefit from the genetic variations in segregating populations. For example, seed yield is a critical characteristic in terms of yield and this value was recorded as maximum 33.4 g in the best parent (female parent), while it was recorded as 83 g in the F_2_ population, more than twice that of the parent (Table 3). Farmers prefer varieties with higher biological yields for forage. In such cases, genotypes that produce more vegetative parts rather than seeds are selected. The maximum biological yield in the F_2_ and F_3_ populations was found to be 237 g and 243 g, respectively, which is almost 3 times higher than the best parent.

**Figure 6:**
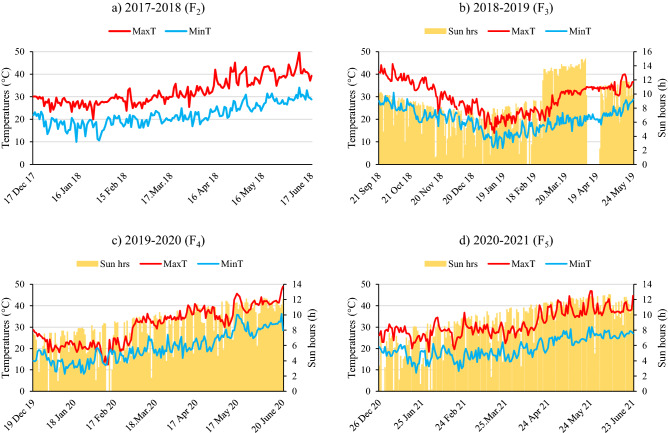
Daily minimum (MinT), maximum (MaxT) temperatures and daily sun hours in the glasshouse in 2017–18 (**a**), 2018–19 (**b**), 2019–20 (**c**) and 2020–21 (**d**).

De novo early progeny was found in segregated populations (Fig. 3, 4 and Table 3). Two progeny flowered in 18 days in F_2_ population (Table 2), whereas progeny from these two progeny flowered in 13 days in F_3_ (Fig. 3 and Table 3). According to the results of the literature review, no study has reported peas that flowered in 13 days. The use of wild peas in crossbreeding studies has been reported by many researchers to increase genetic diversity^51,68–70^, and a wide variation has been reported in the flowering time of pea crop^12–14^. Watts et al.^71^ reported that the flowering time of pea was between 52 and 71 days. Vanhala et al.^15^ determined flowering time in the range of 32.4–87.2 days in a study which researched the adaptation of pea flowering time. Days to flowering was reported to be 31–49, 46–54 and 46–54 days at different years or locations under 18 h photoperiod conditions in crosses *P. sativum* × *P. fulvum* by Jha et al.^60^. In the present study, days to flowering was recorded under short day conditions with daily sun hours (day length) between 2–9 h (Fig. 6). Earliness was induced by long light period under controlled conditions and six generations were advanced via speed breeding in pea^10,11^. If de novo super early lines (Fig. 4) can be grown under suitable conditions, it is considered that 8–10 generations per year can be obtained. The earliest progenies in the F_2_ population were 3 times earlier than the mean of the female parent and 7.5 times earlier than the mean of the male parent in terms of the number of days to flowering and pod setting (Table 3 and Fig. 3). The earliest progeny in the F_3_ population were 3.9 times earlier than the mean of the female parent and 9.5 times earlier than the mean of the male parent in terms of the number of days to flowering and pod setting (Table 3 and Fig. 3). F_2_ and F_3_ populations have shown bimodal distribution for flowering time as a segregation ratio of late to early flowering of 9:7 (Fig. 3a,b). Under short-day conditions, this distribution demonstrated that flowering time is controlled by two genes with duplicate recessive epistasis or complementary gene action. Similar findings on 9:7 distribution for flowering time were reported by in F_2_ population derived from the intraspecific crosses in pea^27^ and chickpea^72^. A bimodal distribution, late and early, was discovered for the flowering node using F_2_ population in pea, and the gene responsible for the late flowering was named *Sn*^73^. *Snsn* gene pair was responsible for the difference between early and late flowering in intraspecific pea crosses^74^. Approximately 20 loci were pointed out to be involved in pea flowering variation^29^, with cultivated alleles usually resulting in early flowering and a decline in photoperiod response^75^. *Hr*, *Sn*, *E,* and *Lf* genes were found to be effective in naturally occurring variation^29^. *Hr*, on the other hand, has just one naturally existing mutant allele, whereas *Sn* has both naturally occurring and induced mutant alleles^76^. In short day conditions, *hr* was reported to be induced early flowering and decreased of the response to photoperiod but not whole loss, while *sn* was indicated to be caused complete daylength insensitivity^22^. Dominant alleles of *E* have been stated to cause early flowering and this effect had complex interactions with other loci^29^. Flowering was inhibited on both long and short days conditions by *Lf*, while accessions having *Lf* gene was inactivated by the nonsense mutation providing extreme earliness^21,22^. The extreme earliness identified in the present study is considered to be due to variants of *Lf* gene.

There is increasing global concern about the impact of climate change on food production, livelihoods, and food security^77,78^. Global warming is anticipated to be one of the biggest hazards for food and it will have adverse effects on agricultural production. It is estimated that the expected world population will be 8 billion by 2030, which will require an increase of 60% in the current food production^79,80^. The vast majority of the world's population lives in cities and given the reasons for migration from rural areas to the city, it is inevitable that the consumption rate will create even more food deficits^78^. According to the Intergovernmental Panel on Climate Change (IPCC), global warming will exceed 1.5 °C by 2030, leading to permanent loss of the most sensitive ecosystems and crisis for vulnerable people and societies in underdeveloped and developing countries^81^. Drought and heat stresses, which create abiotic stressors that significantly reduce the yield of plants, will be the leading causes of global warming in agriculture^82^. Drought and heat effects are expected to increase with climate change and increasing water shortages^83^. Two types of heat stress in agricultural areas are (1) heat shock, which occurs in daytime and lethal temperatures, and (2) moderate heat, which is higher than optimum temperature in daytime or at night^83^. Since pea cultivation is carried out in rainfed areas in some parts of the world, plants suffer from heat stress. Plants exhibit three different mechanisms for heat tolerance, namely, heat escape, heat tolerance and heat avoidance^84^. Heat escape enables plants to quickly complete their life cycle in a short time and under favorable conditions and in this way, plants tend to escape drought due to early maturity^18,85^. Early flowering genotypes in pea play a crucial role in minimizing bottlenecks such as abiotic and biotic stresses and can lower production costs as a result of low input. De novo super early progeny was seen to have the ability to escape from heat stresses, while late flowering progeny were subjected to heat stresses during flowering and pod setting (Fig. 6). Vegetable types were not only high yielding but also free of any known undesirable traits of the wild species, such as pod dehiscence and non-uniform maturity (Fig. 1).

In addition to problems such as global warming, pandemics that cause the death of thousands of people, such as COVID-19, also adversely affect the world^86^. In vulnerable countries where hunger and malnutrition are already common, pandemics such as COVID-19 also pose a threat to food safety and scarcity^87^. Hunger, malnutrition, and unbalanced nutrition affect more than 820 million people worldwide, and approximately 150 million children are negatively affected by an irregular diet^88^. At the beginning of the COVID-19 outbreak, there was an excessive demand for food in the world^89^. Food deficit is a major problem in such pandemics and thus early varieties play an important role in meeting these food needs. Very early pea progeny, which can be obtained in about one month, will be a unique food source able to meet food needs in a short time.

According to the heritability classification of high (> 50%), medium (30–50%) and low (< 30%) described by Guindon et al.^61^, days to flowering and pod setting had high values of narrow sense heritability (Table 3). The high values of narrow sense heritability means that the gain from selection can be achieved by selecting de novo super early progeny. In the present study, days to flowering in de novo super early progeny was fixed with days to flowering of 24–25 days in F_4_ and F_5_ populations (Fig. 4). Guindon et al.^61^ reported that the number of flowering days, number of pods, pod length and number of seeds per pod had high values of heritability, but not plant height. Singh et al.^90^ found high broad sense heritability in pea for days to flowering, and days to pod setting. Since earliness in the present study was seen to have high heritability, success can be achieved in selection in early generations.

Based on principal component analyses (PCAs), earliness including days to flowering and pod setting was related in both F_2_ and F_3_ populations (Fig. 5). Seed yield was correlated with pods per plant, in accordance with the findings of Guindon et al.^61^ and Esposito et al.^91^. According to the available literature, this is the first path analysis on days to flowering in interspecific crosses *P. sativum* × *P. fulvum.* Days to pod setting, plant height, and first pod height had statistically significant direct effects on days to flowering (Table 4). Path analysis performed in the F_2_ population obtained from intraspecific crosses by Singh et al.^90^, showed that plant height and pods per plant had a direct positive effect on seed yield, and a direct negative effect on days to flowering. Similar findings were reported by Singh and Srivastava^92^ and Tiwari et al^93^. In the present study, biological yield and pods per plant had a significant effect on seed yield (Table 4) indicating that seed yield selection should be carried out according to biological yield and pods per plant.

In presenting the results obtained in the present study, the following can be suggested for readers; (1) Not only transgressive segregations but also de novo super early progeny were obtained by interspecific crosses between the cultivated pea (*P. sativum*) and its wild relative *P. fulvum*. (2) Some de novo super early progeny flowered in 24–25 days in both F_4_ and F_5_ generations. (3) The heritability was found to be high in the present study since the earliness trait was little affected by the environment. It can be suggested that traits with high heritability will yield successful results in early generation selection studies. (4) De novo super early progeny obtained from interspecific crosses in the breeding study matured without exposure to heat stress and this progeny can escape from heat stress. (5) It is thought that by using de novo super early progeny obtained in breeding studies, peas with a short vegetation period can be grown without the risk of frost exposure in the northern hemisphere. (6) Green pods and seeds of garden pea are important vegetables in Mediterranean cuisine and they have a high price in markets with earliness whenever the crop is purchased by consumers in autumn. (7) It has also been understood that de novo super early progeny can also be cultivated at high altitude without the risk of frost. (8) It is estimated that much more than six generations in a year under suitable conditions can be obtained via de novo super early progeny. This shows that these de novo super early progeny can be alternative and complementary materials to the speed breeding approach. (9) This study can be considered an example study for the use of wild peas in breeding studies. (10) Useful progeny obtained from crossing wild species with cultivated species reveal the importance of wild species.

## Materials and method

### Parents

ACP 20 is the cultivated genotype of *P. sativum* with mid-early flowering and white flowers, whereas AWP 600 is a wild genotype of *P. fulvum* with late flowering and orange flower color. According to our records, ACP 20 is a landrace grown as vegetable in Antalya, Turkey, while AWP 600 is originated from Turkey and received from USDA GRIN, USA. The plant materials comply with relevant institutional, national and international guidelines and legislations. As seen in Table 1, ACP 20 has large, cream-colored seeds and weight of 42.8 g per 100 seeds. AWP 600 has small, black-colored seeds and weight of 5.4 g per 100 seeds. It is resistant to seed beetle (*Callosobruchus chinensis* L.) and powdery mildew caused by *Erysiphe pisi* DC^37,38^. Both parents, ACP 20 and AWP 600, were grown on the campus of Akdeniz University, Antalya, Turkey (30°38′E, 36°53′N, 33 m above sea level) as a spring-sown crop in 2015.

### Progeny

Reciprocal interspecific crosses *P. sativum* × *P. fulvum* and *P. fulvum* × *P. sativum* were performed. All progeny derived from interspecific crosses *P. sativum* × *P. fulvum* were advanced from F_1_ to F_5_ (Fig. 1), while *P. fulvum* × *P. sativum* interspecific crosses were advanced up to F_1_ since only one F_1_ plant was obtained. *P. fulvum* × *P. sativum* interspecific cross was excluded from the study due to insufficient plants in F_2_ population.

F_1_, F_2_, F_3_, F_4_ and F_5_ populations derived from interspecific crosses *P. sativum* × *P. fulvum* were sown on 14 December 2016, 17 December 2017, 21 September 2018, 19 December 2019 and 26 December 2020, respectively. Harvesting was applied individually as single plants. Each line was advanced as five seeds after F_2_ population. From the F_3_ to the F_5_, each line was advanced as a family consisting of five individuals.

### De novo super-early progeny

Transgressive segregation was defined as the occurrence of progeny with values greater or less than the values of their parents (male and female plants) in segregated generations^35^. These extreme phenotypes are a major mechanism by which extreme or novel adaptations develop^34–36^. Different explanations have been provided to take into account the presence of extreme phenotypes in segregating populations^94,95^. However, de novo super-early progeny is here referred to as progeny with flowering or pod setting and maturity much earlier than the early parent (best parent) in segregating generations.

### Agro-morphological traits

The following phenological traits were recorded in male and female plants and progeny derived from interspecific crosses *P. sativum* × *P. fulvum*. Days to flowering (DF) was recorded as the number of days after germination until the first flowering. Days to pod setting (DP) was recorded as the number of days after germination until the first pod setting. De novo super-early plants were individually harvested and advanced as single plant progeny.

The following agro-morphological traits were recorded on single plant in F_1_ and F_2_, while they were obtained from average of five plants from F_3_ to F_5_. Plant height (PH) and first pod height (FH) were recorded in cm as the height of a plant from the ground to the top of the plant and as the height from ground to the first pod, respectively. Pods per plant (PP) and seeds per pod (SP) were recorded as total number of pods per plant and seeds per pod, respectively. Pod length (PL) was recorded in cm as the length of a pod. Biological yield (BY) was recorded in grams (g) as the total weight of a plant after harvest, while seed yield (SY) was recorded in g as the weight of seeds per plant after harvest. Harvest index (HI) was calculated as a percentage (%), as the ratio of seed yield per plant to biological yield per plant multiplied by 100. For the pod length, three randomly selected pods on each plant were used and the number of seeds per pod trait of the same pods were recorded. Agro-morphological traits were recorded in F_1_, F_2_ and F_3_ progeny and the parents. In the F_4_ and F_5_ populations, days to flowering and days to pod setting were recorded only in early lines and their parents.

### Soil analyses

According to the soil analysis of the experimental field, it was determined that nitrogen and organic matter content were low, pH was found to be as high as 7.62, whereas CaCO_3_ was 26.8%. Although iron and zinc contents were considered to be deficient due to the high pH of the soil, other plant nutrition elements were generally considered to be balanced.

### Daily minimum and maximum temperatures and sun hours in glasshouse

The minimum (MinT) and maximum (MaxT) daily temperatures and daily sun hours (h) in the glasshouse were presented in Fig. 6. The MaxT of the glasshouse were recorded as 50.1 °C, 41.5 °C, 47.3 °C and 46.8 °C for 2017–18, 2018–19, 2019–20 and 2020–21, respectively (Fig. 6).

### Agronomic practices

A drip-irrigation system was used and plants were irrigated with well water at three-day intervals to avoid drought stress. Weed control was performed by hand. Fertilization was not applied because the plants supplied 80% of the nitrogen requirement^55^.

### Heterosis

Hybrid vigor or average heterosis (H_A_) estimated for agro-morphological traits in order to test general combining ability between parents was calculated as:

$$H_{A } \left( \% \right) = \frac{{F_{1} - MP}}{MP} \times 100,$$

where F_1_ is from data of F_1_ plants and MP is the mean of the two parents as mid-parent^66^.

### Narrow-sense heritability

Narrow-sense heritability (${h}^{2}$) performed to explain inheritance of agro-morphological traits was calculated for days to flowering, days to podding and important agro-morphological traits, according to the progeny-parent regression method reported by Poehlman and Sleper^96^.

$$\begin{aligned} & b = { }\sum (X - \overline{X}) \left( {Y - \overline{Y}} \right)/\sum \left( {X - \overline{X}} \right)^{2} , \\ & h^{2} = b \\ \end{aligned}$$

where $b$ is the regression coefficient of the offspring (F_3_) value (Y) to the representing parent (F_2_) value (X). Accordingly, the slope ($b$) of the regression line represents the values of narrow-sense heritability. A similar narrow-sense heritability for agro-morphological traits was given in interspecific and intraspecific crosses in *Cicer* species^66^. Narrow-sense heritability was given as a percentage (%).

### Chi-square analyses for flower color and flowering time

Chi square test (${\chi }^{2}$) was performed to estimate the goodness of fit to the expected ratio in the segregating F_2_ population using a following formula:

$$\chi^{2} = \sum \frac{{\left( {O - E} \right)^{2} }}{E},$$

where *O* and *E* in this formula represent the observed and expected values, respectively^97^.

### Statistical analyses

All data were reported as descriptive statistics including mean, range and standard error values using SPSS 26.0 software (IBM SPSS: Chicago, IL, USA). The regression slope for days to flowering, days to pod setting and agro-morphological traits was calculated using SPSS 26.0 software. Path analysis was also performed to show direct and indirect relationships between days to flowering (earliness) and agro-morphological traits. Principal component analyses (PCAs) were performed using XLSTAT statistical software Version 2016.02 (Addinsoft, Paris).
